# Profiles of lateral violence in nursing personnel of the Spanish public health system

**DOI:** 10.1371/journal.pone.0268636

**Published:** 2022-05-27

**Authors:** David Pina, Maria Vidal-Alves, Esteban Puente-López, Aurelio Luna-Maldonado, Aurelio Luna Ruiz-Cabello, Teresa Magalhães, Bartolomé Llor-Esteban, José Antonio Ruiz-Hernández, Begoña Martínez-Jarreta

**Affiliations:** 1 Department of Socio-sanitary Sciences, University of Murcia, Murcia, Spain; 2 Department of Community Medicine, Information and Health Decisions, School of Medicine, University of Porto, Porto, Portugal; 3 University Institute of Health Sciences—CESPU, Gandra, Portugal; 4 Facultad de las Ciencias de la Naturaleza y, Universidad Nebrija, Madrid, Spain; 5 Department of Nursing, University of Murcia, Murcia, Spain; 6 Servicio de Psicología Aplicada (SEPA), University of Murcia, Murcia, Spain; 7 Department of Pathological Anatomy, Forensic and Legal Medicine and Toxicology, University of Zaragoza, Zaragoza, Spain; 8 Department of Psychiatry and social Psychology, University of Murcia, Murcia, Spain; Universitat de Valencia, SPAIN

## Abstract

**Background:**

Workplace violence in healthcare settings has long been studied in scientific literature, particularly in the nursing profession. Research has explored mostly user violence probably for its high prevalence and impact on health and job satisfaction. Yet this focus may overshadow another dangerous type of workplace violence: coworker violence. Exerted by co-workers with similar status, lateral violence differs from that yielded by a co-worker with a higher rank, known as vertical. This study aims to deepen the knowledge about lateral violence perceived by nurses and its interaction with other variables commonly associated with workplace violence in healthcare: burnout, job satisfaction, and self-perceived health.

**Method:**

A random block sampling was performed, prompting a total sample of 925 nursing professionals from 13 public hospitals located in the southeast of Spain. The sample distribution (mean and standard deviation) and the response percentages according to the study variables of the ad-hoc questionnaire were analyzed and classified with cluster analysis.

**Results:**

Through the cluster analysis, two subgroups were obtained: Cluster 1, composed of 779 participants, with low scores in the variables used for the classification, high levels of both extrinsic and intrinsic satisfaction, low levels of emotional exhaustion and cynicism, and low rates of somatization, anxiety, social dysfunction and depression; and Cluster 2, composed of 115 participants and characterized by moderate-high scores in the variables used for the classification, moderate extrinsic satisfaction, and low intrinsic satisfaction, high emotional exhaustion and cynicism and lower somatization, anxiety, social dysfunction, and depression scores. Excluded cases amounted to 31.

**Conclusion:**

Nursing professionals who experience lateral violence reveal a lower intrinsic satisfaction, feeling less self-accomplished in their job, and less positive work experience. Emotional exhaustion rises as a concerning progressive and long-term outcome of experiencing this type of violence.

## Introduction

Violence in healthcare settings has long and broadly been studied in scientific literature. Research has explored mostly user violence against health personnel probably for its high prevalence (between 50% and 100% of professionals, depending on the country of the study), which is only lesser in western countries [1–3]. Studies have also been influenced by the conceptualization of the term violence, considering it severe when involving physical abuse and moderate to mild, when including insults or humiliation [4–6].

Nonetheless, ensuing Chappell & Di Martino’s model [7], violence in the healthcare setting may relate to multiple factors that go way beyond professional-user variables. These authors propose five wide groups of variables that may influence workplace violence. The first is focused on user-related factors, evincing males, younger people, and patients with hospital admission against their will or with a diagnosis of psychosis. The second is dedicated to the professionals’ features. In this perspective, the more intense type of violence (physical) is more frequently received by male professionals, being young of age or in the profession and the type of contract or shift detained, are factors that seem to influence the exposure to violence. The third group of variables includes environmental factors such as the size of the hospital and the type of department or area of work. The fourth is related to patient care, especially the clear communication of treatment abandonment or cognitive impairment, that are central in this group. Finally, the fifth group refers to social or interactive factors: the more interaction there is between professional and patient, the higher the risk of conflict [1–3, 8, 9].

Although this model encompasses a great deal of the complexity of workplace violence perceived by health professionals, it seems to be somehow limited to user violence. This focus withdraws attention from other possible sources of workplace violence, such as lateral/horizontal violence or from a co-worker with a similar rank, and vertical violence, which is the one received from a co-worker with a higher rank [10].

The International Labour Organization defines workplace violence in the healthcare setting as any *“intentional work-related abuse*, *assault and threats towards professionals*, *in their workplace*, *including physical and psychological violence*” [7, 11]. When referring to lateral violence, it forcibly includes violence between colleagues which can take different forms: personal, work-related, and social [10]. The first is characterized by behaviors such as verbal aggression, spreading gossip, persistent criticism, playing practical jokes, intimidation, attacks on the victim’s private life, humiliation, acts of contempt, emotional abuse, and social exclusion [10, 12, 13]. The second includes assigning unreasonable deadlines and unmanageable workload or no tasks at all, or even meaningless ones, controlling or manipulating information and work conditions, besides excessively monitoring the targeted worker [10, 12, 13]. The third includes being ignored, undervalued and impeded from getting training or research [10].

One of the most severe consequences of this phenomenon is burnout syndrome, defined as a condition caused by stressful workplaces that encompasses emotional exhaustion, depersonalization and personal accomplishment [14]. Burnout is a frequent outcome in violent workplaces due to the stressful environment to which professionals are exposed on a daily basis [15]. Its prevalence rates are high in the healthcare professions [16] and strongly affect nurses, physicians and helping staff [17, 18]. Strong predictors of this syndrome include unrealistic demands, extreme workload and working shifts or night shifts only [19] as well as the perceived poor quality of working conditions [20]. Specially challenging times such as the Covid-19 pandemic have severely increased the impact of these risk factors due to a notably higher workload [21].

Workplace violence has been associated to poorer physical and mental health outcomes [22, 23] and unhealthy behaviours [24]. The most frequent effects of workplace violence on mental health include anxiety and depressive symptoms, acute stress, burnout syndrome, suicidal risk and post-traumatic stress disorder [25, 26]. A recent systematic review found an overall significant relationship between workplace violence and increased risk of sleep disorders, with verbal violence posing the most deleterious effects [27] with demonstrated overall impaired work function in nursing personnel [23] and high costs both to professionals and the healthcare system [28].

The present study aims to address lateral violence in healthcare settings and envisions to analyze its interactions with variables that are classically associated with workplace violence such as burnout, job satisfaction, and perceived violence. The specific objectives are: (a) to derive empirical subgroups of nursing professionals using exploratory cluster analysis; (b) to investigate the subgroups created according to their exposure to lateral violence in the workplace; (c) to observe possible differences in the profiles obtained according to possible consequences related to lateral violence (extrinsic and intrinsic satisfaction, emotional exhaustion, professional efficacy, cynicism, somatic symptoms, anxiety and insomnia, social dysfunction and depression).

## Material and methods

### Sample

A random block sampling was performed, prompting a total sample of 950 nursing professionals from 13 public hospitals located in the southeast of Spain. Of these, 6 hospitals were considered large (200 beds capacity or more) and 7 were considered medium or small (200 bed capacity or less).

Considering the sample’s characteristics (Table 1), the age of participants ranged from 30 to 50 years, with a mean age of 39.43 years (SD = 9.65). Most were women (77.8%) and married or cohabiting (63.2%). Regarding work characteristics, 54.3% were in the nursing profession for 0 to 5 years (mean time was 14.02 years) and at least 54% were in the same job position for the last 5 years (mean 7.31 years, SD = 8.35). From the studied sample, 20.3% worked in surgery, 17% in internal medicine, 14.3% in an emergency, 6.9% in day-care, 5.5% in mental health, and 14.8% in other facilities.

**Table 1:** Sociodemographic and work-related variables.

Variables	N	%
**Sex**		
Female	720	77.8
Male	199	21.5
Missing data	6	0.6
**Marital status**		
Single	290	31.4
Married or cohabiting	585	63.2
Divorced, separated and/or widow	45	4.9
Missing data	5	0.5
**Age**		
<30	137	14.8
30–50	586	63.4
+50	153	16.5
Missing data	49	5.3
**Length of time in the profession (years)**		
<5	502	54.3
6–11	208	22.5
12–20	120	13.0
>20	70	7.6
Missing data	25	2.7
**Length of time in the job position (years)**		
<1	120	13.0
1–5	382	41.3
6–10	180	19.5
11–15	84	9.1
>15	134	14.5
Missing data	25	2.7
**Units**		
Surgery	188	20.3
Mother and child healthcare	101	10.9
Internal medicine	161	17.4
Emergencies	132	14.3
External Consultations/ Outpatient	64	6.9
Mental Health	51	5.5
Other	137	14.8
Missing data	91	9.8
**Type of hospital**		
Large	761	82.3
Medium or small	164	17.7

### Variables and instruments

A 76-item protocol was built with items about the sociodemographic variables of the respondents (age, sex a marital status) and work-related variables (length of time in the profession, length of time in the current job position, hospital size, and type of unit).

The division of variable for the present study, were organized into the following: (a) Clustering variables, as necessary to apply to cluster analysis and form the subgroups, bearing in mind *personal lateral violence*, *social lateral violence*, *and work-related lateral violence*; (b) Profiling variables, for comparison purposes with prior analysis resulting subgroups, regarding *extrinsic satisfaction*, *intrinsic satisfaction*, *emotional exhaustion*, *professional efficacy*, *cynicism*, *social dysfunction*, *somatic symptoms*, *anxiety*, *and depression*. These variables were measured with the following instruments:

#### Clustering variables

*Personal lateral violence*, *social lateral violence*, *and work-related lateral violence*, measured with the Health Workers Aggressive Behavior Scale–CoWorkers and Superiors (HABS-CS). The HABS-CS was created by Waschgler, et al. [8] and assesses incivility and hostile behavior perceived by health professionals by co-workers. It encompasses 10 items with 6 response options (1 = never to 6 = daily) grouped in the three variables studied. The original study presents an internal consistency of .82 for the personal scale, .79 for the social scale, and .72 for the work-related scale, with a total Cronbach Alfa of .864. For the present study, the total liability of .87 was met for the total scale and .82, .82, and .71 in the personal, social, and labor factors, respectively.

#### Profiling variables

The Maslach Burnout Inventory-General Survey (MBI-GS), created by Schaufeli et al. [29] was the tool elected to measure *Emotional exhaustion*, *professional efficacy*, *and cynicism* were measured with. In this study we used the Spanish version, translated and validated by Gil-Monte [30]. The 16 items are organized within the three variables above indicated, grouped in 5, 5, and 6 items, each. Responses range from 0 (never) to 6 (always). Gil-Monte’s study presents an internal consistency of .83 for emotional exhaustion, .72 for professional efficacy, and .73 for cynicism [30] while the present study, found the following Cronbach Alphas, respectively: .85, .85, and .70.

*Intrinsic satisfaction and extrinsic satisfaction*, measured with the Overall Job Satisfaction scale (OJS). The OJS, first built by Warr, Cook, and Wall [31], was adapted to Spanish by Pérez and Fidalgo [32], which is the version used herein. It encompasses 15 items organized in the two above-mentioned subscales. Answers may be positioned from 1 (very unsatisfied) to 7 (very satisfied). The original study presented an internal consistency of .85 to .88 for extrinsic factors and .74 to .78 to intrinsic. The present study yields .84 to the first and .70 to the later.

*Anxiety*, *insomnia*, *social dysfunction*, *somatic symptoms*, *and depressive symptoms*, were measured by the General Health Questionnaire (GHQ-28). This tool was developed by Goldberg and Hillier [33] for the evaluation of general health. Its Spanish version, used in the current study, was adapted by Lobo, Pérez-Echevarría, and Antral [34], including 28 items grouped in the four variables included in this study. Answers are provided in four options from zero to three (0–3) of lower to higher intensity. The original study’s internal consistency was .78 for Somatic GHQ, .85 for Anxiety GHQ, .75 for Dysfunction GHQ, and .78 for Depression GHQ. The present study yielded .79 for both Somatic GHQ and Anxiety GHQ, .71 for Dysfunction GHQ, and .78 (Depression GHQ).

### Procedure

For sampling purposes, the authors contacted the directors and supervisors of the participant hospitals to provide detailed information about the present study and its goals. Upon acceptance, a meeting was arranged with the supervisors of the different units (as fellow researchers) during which the study protocols were delivered. These included an informative note, the above-mentioned scales, and instructions regarding its fulfilment, informed consent to participants (obtained after reading information and filling the protocol’s questionnaire), and delivery to the research team in a sealed envelope. A code was ascribed to each worker and protocols were randomly assigned to 50% of the sample. The protocol was delivered by fellow researchers who later managed its reception, in a sealed envelope without identification in a maximum deadline of two weeks. Protocols undelivered during this time length were considered lost. A response rate of 70.48% was obtained. The present study, designed under STROBE guidelines, received approval of the Ethics Committees and directive boards of each hospital. The present study was approved by the Research Ethics Committee of the authors’ home University (ID: 3555/2021). The authors disclose no conflict of interest.

### Data analysis

Data analysis for the current study was performed using SPSS version 25. The sample distribution (mean and standard deviation) and the response percentages according to the study variables of the ad-hoc questionnaire were analysed. We used the Pearson correlation test to complete the analysis of the relationship between the scales used and a cluster analysis was used to empirically derive subgroups of nursing professionals. The analysis was performed following these three steps: (1) determining of the number of clusters found ideal. The method followed by Aguerrevere et al. [35] was put into practice using the SPSS two-step auto-clustering analysis. This procedure selects the ideal total of clusters via which it takes the highest value of the ratio of distance measures (RDM), the lowest value of Schwarz’s Bayesian Criterion (BIC) and the highest BIC change; (2) the cluster analysis using the K-means procedure with the clustering variables as well as the best possible number of clusters found in the prior analysis; and (3) cross-validation.

Subsequently, we analysed the differences in the profiling variables using Student´s *t*-test and performed a discriminant analysis on the variables used to form the clusters, stepwise. We thus assessed if the mentioned group of variables allows predicting group membership.

The clustering variables were standardized as Z-scores, because of differences in the results’ format. Consequently, the Z-scores were transformed into the original values of each scale in order, thus enabling both presentation and understanding of the produced results.

## Results

It was possible to observe that a minimum of 59.2% was exposed to violence from a co-worker at least once in the last year. Specifically, 51% of the sample was exposed to lateral violence of personal nature (e.g., “*Some co-workers spread false rumors about me*”), 37.3% of social nature (e.g., “*Some co-workers have stopped talking to me*”), and 21.3% work-related (e.g., “*Some co-workers deliberately accuse me of other people’s mistakes*”).

### Lateral violence and external correlates

Concerning the relationship between social lateral violence and possible consequences in health professionals (Table 2), the Pearson correlations obtained confirm that personal lateral violence is significantly negatively correlated to extrinsic (r = -.18, p = .01) and intrinsic satisfaction (r = -.19, p = .01) as well. In contrast, a significant positive correlation was found to emotional exhaustion (r = .28, p = .01), cynicism (r = .21, p = .01), somatic symptoms (r = .21, p = .01), anxiety (r = .24, p = .01), social dysfunction (r = .07, p = .05) and depression (r = .20 p = .01). No significant correlation was met to perceived professional efficacy.

**Table 2:** Correlations between social lateral violence and possible consequences in health professionals.

	1	2	3
**1.** HABS Personal	1		
**2.** HABS Social	.55**	1	
**3.** HABS Work-related	.50**	.57**	1
**4.** Extrinsic Satisfaction	-.18**	-.18**	-.17**
**5.** Intrinsic Satisfaction	-.19**	-.20**	-.22**
**6.** Emotional exhaustion	.28**	.20**	.17**
**7.** Professional Efficacy	-.04	-.05	-.04
**8.** Cynicism	.21**	.18**	.17**
**9.** Somatic symptoms	.21**	.17**	.15**
**10.** Anxiety and insomnia	.24**	.16**	.16**
**11.** Social dysfunction	.07*	.12**	.16**
**12.** Depression	.20**	.20**	.18**

* = p<0.05

** = p<0.01

Concerning the social forms of lateral violence, a significant negative correlation emerged with satisfaction in both its scopes, being the r values of .18 (p = 0.01) to extrinsic satisfaction and 0.20 (p = .01) to intrinsic satisfaction. A significant positive correlation to emotional exhaustion (r = .20, = .01), cynicism (r = .18, p = .01) and the variables measured by GHQ-28: somatic symptoms (r = .17, p = .01), anxiety (r = .16, p = .01), social dysfunction (r = .12, p = .01) and depression (r = .20, p = .01) was encountered. No relation was found to professional efficacy (r = -.05, p = .05).

Lastly, while studying the relationship between the work-related lateral violence and the hypothesized consequences, a negative correlation was found to both extrinsic (r = -.17, p = .01) and intrinsic satisfaction (r = -.22, p = .01). On the other hand, it was found to be positively correlated to emotional exhaustion, cynicism (r = .17, p = .01 for both), anxiety and social dysfunction (r = .16, p = .01), somatic symptoms (r = .15, p = .01), and depression (r = .18, p = .01). Again, no correlation was found though to professional efficacy (r = -.04, p = .05).

### Cluster analysis

A two-step cluster analysis was performed, with a logarithm of likelihood and self-clustering method on the entire sample, using the clustering variables as input. The results obtained point to the two clusters’ solution as the most appropriate, with the highest BIC change (BIC = -932.27, RDM = 5.88, BIC change = 308.27). The three and four cluster solutions provided a lower RDM and a higher BIC, besides a BIC change (BIC = -125.53, RDM = 1.30, and BIC = -84.19, RDM = 2.06 respectively). The *silhouette* cohesion and separation measure confirmed the cluster quality as good (.82).

Subsequently, we conducted a K-means clustering analysis using the three variables depicted in Table 3. The participant’s distribution was: Cluster 1 with 779 participants (84.2%) and Cluster 2 with 115 participants (12.4%). Excluded cases amounted to 3.4% (31).

**Table 3:** Means, SD and ANOVAs of the clustering and profiling variables.

	Cluster 1		Cluster 2		*t*	*p*	*d*	IC 95%	
	*M (SD)*		*M (SD)*					Inf	Sup
Personal Lat. Violence	4.98	(1.34)	11.86	(3.58)	-22.15	.00	3.49	-7.53	-6.30
Social Lateral violence	3.31	(.81)	6.29	(3.29)	-10.45	.00	1.83	-3.54	-2.41
Work-related Lat. Viol.	3.21	(.67)	5.19	(2.90)	-7.86	.00	1.40	-2.47	-1.47

Abbreviations: *M*: Mean; *SD*: Standard Deviation; *d*: Cohen´s *d* effect size–Cohen’s *d*; 95% IC Inf Sup: confidence interval of 95% with upper and lower limits.

Cluster 1 was composed of workers scoring low in all three variables used to form clusters. The mean scores obtained for personal lateral violence were 4.94 (SD = .36), 3.31 for social lateral violence (SD = .81), and 3.21 for work-related lateral violence (SD = .67). On the other hand, Cluster 2 was composed of workers with a higher exposure, displaying mean scores of 11.86 in personal lateral violence, 6.29 in social lateral violence (SD = 3.29), and 5.19 in work-related lateral violence (SD = 2.90).

Concerning scores obtained for each group in the profiling variables, significant differences were found for all except professional efficacy [*t*(195) = 1.77; p = .34; d = .09]. Social dysfunction [*t*(926) = -14.68; p = .00; d = 1.22], emotional exhaustion [*t*(167) = -5.98; p = .00; d = .64] and intrinsic satisfaction [*t*(172) = 5.41; p = .00; d = .55] evinced the highest effect magnitude.

Cluster 1 presented high levels of both extrinsic and intrinsic satisfaction (M = 30.76, SD = 6.46 and M = 26.31, SD = 6.86, respectively), revealing, at the same time, low levels of emotional exhaustion and cynicism (M = 13.70; SD = 4.99 and M = 11.73; SD = 4.86). It also exhibits low rates of poorer mental health indicators such as somatization (M = 12.53, SD = 3.57), anxiety (M = 12.04, SD = 3.93), social dysfunction (M = 13.53, SD = 1.77), and depression (M = 8.02, SD = 2.13). Contrariwise, Cluster 2 showed moderate extrinsic satisfaction (M = 27.61, SD = 7.57) and a low intrinsic satisfaction (M = 22.44, SD = 7.74), and high emotional exhaustion and cynicism (M = 17.04, SD = 6.12 y M = 14.08, SD = 5.33). But it also unveils higher scores of somatization (M = 13.97, SD = 4.20), anxiety (M = 13.95, SD = 4.38), social dysfunction (M = 15.94, SD = 2.64) and depression scores (M = 9.03, SD = 3.19). Differences between groups are depicted in Table 4.

**Table 4:** Means, SD and ANOVAs of the clustering and profiling variables.

	Cluster 1		Cluster 2		*t*	*p*	*d*	IC 95%	
	M (SD)		M (SD)					Inf	Sup
Extrinsic Satisfaction	30.76	(6.46)	27.61	(7.57)	4.53	.00	.47	1.78	4.52
Intrinsic Satisfaction	26.32	(6.86)	22.45	(7.74)	5.41	.00	.55	2.57	5.15
Emotional Exhaustion	13.71	(4.99)	17.04	(6.13)	-5.98	.00	.64	-4.44	-2.24
Professional Efficacy	28.13	(8.67)	27.38	(8.02)	.96	.32	.09	-0.82	2.33
Cynicism	11.74	(4.86)	14.08	(5.34)	-5.06	.00	.47	-3.25	-1.43
Somatization	12.53	(3.58)	13.97	(4.20)	-3.73	.00	.39	-2.20	-0.68
Anxiety and insomnia	12.05	(3.93)	13.96	(4.39)	-5.08	.00	.47	-2.64	-1.17
Depression	8.03	(2.13)	9.04	(3.20)	-3.51	.00	.42	-1.58	-0.44
Social Dysfunction	13.53	(1.77)	15.94	(2.65)	-14.68	.00	1.22	-2.73	-2.08

Abbreviations: lat.: lateral; viol.: violence; M: Mean; SD: Standard Deviation; d: effect size/magnitude?–Cohen’s d; 95% IC Inf Sup: confidence interval of 95% with upper and lower limits.

### Discriminant analysis

A discriminant analysis was performed to validate the groups. The following cluster variables were used: Personal lateral violence (Wilk λ = .36, p = .000), Social lateral violence (Wilk λ = .65, p = .000), and Work-related lateral violence (Wilk λ = .76, p = .000).

The statistical analysis resulted in a function with significant results (X^2^ = 984.89, p = .00, Wilk λ = .33). Function 1 accounts for 100% (canonical function correlation = .81) of the relationship between variables and the clustering generated subgroups. As depicted in Table 5, the strongest relationship to Function 1 is observed in Personal lateral violence. Finally, the confirmation that the model was able to classify accurately 96.5% of the three subgroups (100% of cluster 1 and 77% of cluster 2) was obtained by cross-validation.

**Table 5:** Discriminant function analysis coefficients for independent variables and clusters obtained through the k-mean method.

	Function 1	
Variables	Non standardized coefficients	Correlation coefficients
Personal lateral violence	.46	.93*
Social lateral violence	.23	.50
Work-related lateral violence	.04	.38

*Highest absolute correlation between each variable and any discriminant function

## Discussion

The present study had the main goal of finding which characteristics were observed in nursing professionals depending on the lateral violence perceived. With a sample of nurses, mostly young of age but also in the profession or current job and largely female, the current results indicate that personal, social, and work-related violence are significantly negatively correlated to both extrinsic and intrinsic satisfaction and positively correlated to dimensions of burnout and poorer health quality. This is similar to what is widely observed in other workplace violence types, such as user violence [1–3].

It is understood that the correlation between the exposure to lateral violence and the two variables of job satisfaction, intrinsic and extrinsic satisfaction, is of the utmost importance. Evidence shows that these two variables have a significantly high impact on overall job satisfaction [36–38]. In general, these findings show that those who suffer from this type of violence may experience lower intrinsic satisfaction concerning factors linked to workers’ self-training and education, in the sense of a need for positive sensations and of self-accomplishment in the workplace [36]. In this sense, a low intrinsic satisfaction entails a perception by the professional of low responsibility attribution, low decision-making opportunities, and that his/her skills are not appropriately used and developed or duly valued, having a low accomplishment sensation [39].

Similarly, extrinsic satisfaction, seen as a relationship with organizational variables, is also impacted. This implies that the professional develops a generally more negative view of his/her job and its related features, such as salary, company policies, shifts, and work schedules or workload approval [38]. The association between these variables in the current study has also confirmed the findings of other ER-based studies, such as Swafford’s [40], in which 91.7% of respondents stated that lateral violence decreases their job satisfaction, with 53.3% pondering transferring to another unit, hospital, or leaving their job.

Likewise, a relationship was found between lateral violence and both burnout-related and mental health-related variables. Those who suffer from this type of violence feel more exhausted emotionally and develop more anxious-depressive and somatic symptomatology. In the same way, they may internalize, self-desensitize, or fall into lethargy in the face of various aspects of their job (cynicism) [30] and of their functioning or social interaction abilities (social dysfunction) [30].

In an inductive thematic analysis by Krut et al [41], LV is proven to be destructive to mental health and a strong reason leading new nurses to abandon their profession as the emerging subthemes were the *cycle of violence*, *nurses eating their young*, *shame*, *drowning*, *isolation and vulnerability*.

Lateral violence is disruptive and inadequate behavior in the workplace that is perceived by a high percentage of nurses in their work, somewhat due to their demanding and highly supervised environment [42, 43]. It is a common cause of stress and depression, and, besides leading nurses to consider leaving their jobs [41, 44–46] they negatively impact patient attention and care in case they stay in the job [41, 47].

Data resulting from the cluster analysis are coherent with the correlational analysis priorly performed. It further allowed the creation of differentiated subgroups. The first, named Cluster 1, evinces a low exposure to all three types of lateral violence and is characterizable as a profile with moderate-high satisfaction, with a mild mental health variation (subclinical), and a moderate-high alteration of variables related to burnout.

Incivility in the workplace concurs with poorer health and is a common source of burnout for nurses but this impact is often mitigated by social support and resilience [48]. Other studies with a broader sample of professionals, also point to the mediating role of resilience when facing harassment in the workplace [49], and argue that the more resilient the targeted nurses are, the more stress they endure, without exhibiting the common alert signs.

On the other hand, the second profile, named cluster 2, whose individuals reported much higher exposure to all three types of lateral violence, evinces similar features to the first profile, but display lesser job satisfaction, more severe alterations to general health (which is still kept at a subclinical level) and higher score on burnout-related variables, especially emotional exhaustion.

When comparing both profiles, all variables obtained relevant effect magnitudes, except for professional efficacy. The highest was observed in emotional exhaustion and social dysfunction, followed by cynicism, anxiety, and insomnia. It is especially relevant that, although Cluster 2 presents higher scores than Cluster 1, in the profile variables in general, the only variable that displays a definite severity variation is emotional exhaustion. This variable changes from moderate-high in Cluster 1 to high in Cluster 2. This may be an indicator of the gradual increase of the negative consequences’ impact on nurses who suffer lateral violence.

This current finding is consistent with that of prior research. Kim et al. [18] studied the relationship between workplace bullying and professional quality of life, burnout and turnover intent in a sample of clinical nurses to find a significant relationship between the first variable and all subdomains of burnout, especially emotional exhaustion. This last variable was signalled by Leiter and Maslach [50] as *the core burnout domain directly affected by workplace bullying”*. Wolf et al. [51] achieved identical results in a qualitative study in which emergency nursing personnel identified lateral violence in the source of emotional and mental exhaustion. In the mentioned study, the emergency nurses identified that emotional and mental exhaustion where a result of lateral violence and had a cyclic progression. These authors stated that “*Our participants described lateral violence as a circular phenomenon that is both a cause and effect of fatigue (…) fatigue led to bullying*, *which in turn led to more fatigue*” (p.3). The main causes pointed out were the inadequate work conditions in a phenomenon that they described as “*competitive nursing*”. This concept leads to the idea that competitive effort and an excessive workload is seen by nurses as what makes them more valuable and well-considered in decision-making moments.

The incivility and bullying among nurses remain widespread as a form of rite of passage is reinforced in nurses training [52] and the *toxic nursing culture* is particularly felt during the first year of nursing practice with a deleterious impact on nurses’ mental health [41].

Yet, this is not impossible to prevent. Research suggests that simulation and role-plays against workplace violence, during academic training, are promising tools [53, 54]. Considering the hazards of workplace violence to health professionals and to the healthcare services, organizational policies became mandatory in developed countries in order to prevent it and its consequences to psychological health [55, 56]. Preventive programs and health promoting benchmarking may prevent workplace violence from happening or at least detecting its occurrence in an early stage [56, 57].

### Study limitations

The present study has limitations. Firstly, it was not possible to apply the questionnaire to 100% of professionals of all participant centres which may influence the obtained results. The high work demand of the studied sample is well known, so it was necessary to reduce the extent of the evaluation and exclude variables that might help a better understanding of the phenomenon, as user violence, for example. Finally, data were collected before the COVID-19 pandemic. As such, certain variables that may have changed due to organizational shifts occurred in the Spanish public health system.

## Conclusions

The analysis of conglomerates exhibits different profiles and symptomatic heterogeneity, which sustains the influence of workplace violence as posing a risk to the wellbeing of healthcare professionals. The comparison between groups shows that professionals with higher exposure to lateral violence feel lesser job satisfaction and more deleterious effects on their psychological and physical health.

Facing these results, it is possible to conclude that these results allow the identification of a professional profile that can benefit from prevention and intervention programs that contribute to improving the work environment’s quality and the professional’s wellbeing.
